# Cancer-associated fibroblasts (CAFs) gene signatures predict outcomes in breast and prostate tumor patients

**DOI:** 10.1186/s12967-024-05413-2

**Published:** 2024-06-27

**Authors:** Marianna Talia, Eugenio Cesario, Francesca Cirillo, Domenica Scordamaglia, Marika Di Dio, Azzurra Zicarelli, Adelina Assunta Mondino, Maria Antonietta Occhiuzzi, Ernestina Marianna De Francesco, Antonino Belfiore, Anna Maria Miglietta, Michele Di Dio, Carlo Capalbo, Marcello Maggiolini, Rosamaria Lappano

**Affiliations:** 1 Department of Pharmacy, Health and Nutritional Sciences, University of Calabria, Rende, 87036 Italy; 2 Department of Cultures, Education and Society, University of Calabria, Rende, 87036 Italy; 3 Department of Medicine and Surgery, University of Enna “Kore”, Enna, 94100 Italy; 4 Endocrinology, Department of Clinical and Experimental Medicine, University of Catania, Garibaldi-Nesima Hospital, Catania, 95122 Italy; 5 Breast and General Surgery Unit, Annunziata Hospital Cosenza, Cosenza, 87100 Italy; 6 Division of Urology, Department of Surgery, Annunziata Hospital, Cosenza, 87100 Italy; 7 Complex Operative Oncology Unit, Annunziata Hospital Cosenza, Cosenza, 87100 Italy

**Keywords:** Cancer-associated fibroblasts (CAFs), Gene signature, Breast cancer, Prostate cancer, K-means algorithm

## Abstract

**Background:**

Over the last two decades, tumor-derived RNA expression signatures have been developed for the two most commonly diagnosed tumors worldwide, namely prostate and breast tumors, in order to improve both outcome prediction and treatment decision-making. In this context, molecular signatures gained by main components of the tumor microenvironment, such as cancer-associated fibroblasts (CAFs), have been explored as prognostic and therapeutic tools. Nevertheless, a deeper understanding of the significance of CAFs-related gene signatures in breast and prostate cancers still remains to be disclosed.

**Methods:**

RNA sequencing technology (RNA-seq) was employed to profile and compare the transcriptome of CAFs isolated from patients affected by breast and prostate tumors. The differentially expressed genes (DEGs) characterizing breast and prostate CAFs were intersected with data from public datasets derived from bulk RNA-seq profiles of breast and prostate tumor patients. Pathway enrichment analyses allowed us to appreciate the biological significance of the DEGs. K-means clustering was applied to construct CAFs-related gene signatures specific for breast and prostate cancer and to stratify independent cohorts of patients into high and low gene expression clusters. Kaplan-Meier survival curves and log-rank tests were employed to predict differences in the outcome parameters of the clusters of patients. Decision-tree analysis was used to validate the clustering results and boosting calculations were then employed to improve the results obtained by the decision-tree algorithm.

**Results:**

Data obtained in breast CAFs allowed us to assess a signature that includes 8 genes (*ITGA11*, *THBS1*, *FN1*, *EMP1*, *ITGA2*, *FYN*, *SPP1*, and *EMP2*) belonging to pro-metastatic signaling routes, such as the focal adhesion pathway. Survival analyses indicated that the cluster of breast cancer patients showing a high expression of the aforementioned genes displays worse clinical outcomes. Next, we identified a prostate CAFs-related signature that includes 11 genes (*IL13RA2*, *GDF7*, *IL33*, *CXCL1*, *TNFRSF19*, *CXCL6*, *LIFR*, *CXCL5*, *IL7*, *TSLP*, and *TNFSF15*) associated with immune responses. A low expression of these genes was predictive of poor survival rates in prostate cancer patients. The results obtained were significantly validated through a two-step approach, based on unsupervised (clustering) and supervised (classification) learning techniques, showing a high prediction accuracy (≥ 90%) in independent RNA-seq cohorts.

**Conclusion:**

We identified a huge heterogeneity in the transcriptional profile of CAFs derived from breast and prostate tumors. Of note, the two novel CAFs-related gene signatures might be considered as reliable prognostic indicators and valuable biomarkers for a better management of breast and prostate cancer patients.

**Supplementary Information:**

The online version contains supplementary material available at 10.1186/s12967-024-05413-2.

## Introduction

Hormone-dependent cancers, including breast, endometrium, ovary, prostate, testis, thyroid, and osteosarcoma, share the hormone-driven stimulation of cell proliferation and subsequent somatic mutations as a main mechanism of carcinogenesis [1]. In particular, breast and prostate tumors rank as the most commonly diagnosed malignancies among women and men, respectively, worldwide [2]. Although improvements in understanding the molecular mechanisms implicated in the progression of breast and prostate cancers have contributed to their better management over the last decade, they remain the second leading cause of cancer death for females and males, respectively [2, 3].

Epidemiological and experimental data suggested a main role for estrogen and estrogen-like molecules in the pathogenesis of breast cancer [4]. The pivotal role of estrogen in the progression of this malignancy is underscored by data showing that approximately 70% of all breast tumors express the nuclear estrogen receptor (ER), particularly the isoform α (ERα). By acting as a ligand-activated transcription factor, ERα mediates the proliferation and invasion of ER-expressing breast tumor cells [5, 6]. Moreover, it has been reported that estrogen may trigger stimulatory signals in breast cancer through membrane receptors unrelated to the nuclear ERs [7, 8]. The classification of breast tumors is based on the status of ER and further prognostic biomarkers, including progesterone receptor (PR), human epidermal growth factor receptor (HER2), and the proliferation marker Ki-67 [9]. The aforementioned factors play a mandatory role in dictating precision treatment [10]. Regardless of the advancement in the development of diagnostic methods and innovative therapeutics that increased the survival rates of women diagnosed with breast cancer, the occurrence and mortality rates of breast cancer patients are significantly increasing worldwide [11].

Prostate cancer is a complex and heterogeneous disease. The risk increases with age and is strongly associated with a family history of any cancer as well as the accumulation of somatic mutations in prostate cells over a patient’s lifetime [12]. One of the most investigated and therapeutically targeted oncogenes in prostate cancer is the androgen receptor (AR), which is frequently amplified and/or mutated in metastatic diseases whereby mediates the androgen-dependent transcription of growth-related genes toward prostate tumorigenesis [13, 14]. In addition to AR, prostate cancer is classified based on the stage and the grade (Gleason score) that, along with histopathological and molecular features and patient characteristics, drive the appropriate clinical management of prostate cancer [15, 16]. In particular, taking advantage of the screening of the prostate-specific antigen, most prostate tumors are diagnosed early and managed successfully. Nevertheless, approximately 15% of men with localized disease exhibit a high risk of developing fatal disease recurrence [17].

The tumor microenvironment (TME) has a crucial role in the development and progression of solid tumors, including breast and prostate cancer [18–20]. Beyond the extracellular matrix (ECM) components, TME includes a variety of interlinked cells, such as mesenchymal cells, endothelial cells, pericytes, and immune cells [21, 22]. In particular, cancer-associated fibroblasts (CAFs), which are the most abundant cell types within the breast and prostate TME, are recognized as active promoters of the progression of both malignancies [23–25]. Specifically, CAFs can endorse tumor occurrence, growth, metabolic reprogramming, angiogenesis, invasion, and metastasis by releasing chemokines, cytokines, growth factors, and matrix-degrading enzymes [26]. The dynamic and bidirectional interactions that occur between CAFs and cancer cells are also engaged in the suppression of immune cells and the development of tumor resistance to chemotherapy, radiotherapy, and targeted therapy [26, 27]. According to this evidence, high-throughput RNA-sequencing (RNA-seq) studies have described the prognostic significance of CAFs and their biomarkers in diverse tumors, including breast and prostate cancer [28–31]. Nevertheless, the high heterogeneity of CAFs concerning their origin, expression of biomarkers, and functions [32], makes hard the comprehensive evaluation of the prognostic impact of CAFs.

Here, we identified two CAFs-related gene signatures for breast and prostate cancer patients, respectively, by integrating RNA-seq data from both primary CAFs and cancer datasets. By providing valuable insights on the transcriptomic landscapes of breast and prostate CAFs and their associated prognostic values, our findings might help to optimize risk stratification and provide new insights into individual treatments for these malignancies.

## Methods

### Cell cultures

Breast and prostate CAFs were isolated, cultured, and characterized as previously described [33], from 20 invasive mammary ductal carcinomas and 20 prostate adenocarcinomas, respectively, and pooled for the subsequent studies. Briefly, specimens were cut into 1–2 mm diameter pieces, placed in a solution containing 400 IU collagenase, 100 IU hyaluronidase, 10% fetal bovine serum (FBS), antibiotics and antimycotics (Thermo Fisher Scientific) and incubated overnight at 37 °C. After centrifugation at 90 × g for 2 min, the supernatant containing fibroblasts was centrifuged at 485 × g for 8 min; the pellet obtained was suspended in DMEM/F-12 with phenol red (supplemented with 10% FBS and 100 µg/ml penicillin/streptomycin). CAFs were then expanded into 10-cm Petri dishes and stored as cells passaged for three population doublings within a total of 7 to 10 days after tissue dissociation. We used CAFs passaged for up to 10 population doublings for the experiments to minimize clonal selection and culture stress, which could occur during extended tissue culture. CAFs were maintained in DMEM/F-12 with phenol red (supplemented with 10% FBS and 100 µg/ml penicillin/streptomycin) and grown in a 37 °C incubator with 5% CO_2_.

### Immunofluorescence studies

CAFs were characterized by immunofluorescence with human anti- fibroblast activation protein α (FAP) antibody (H-56), which was also used to assess fibroblast activation, and human anti-cytokeratin 14 (LL001) (Santa Cruz Biotechnology, DBA, Milan, Italy). Briefly, cells were grown on a cover slip, next were fixed in 4% paraformaldehyde in phosphate buffered saline (PBS), permeabilized with 0.2% Triton X-100, washed 3 times with PBS and incubated at 4 °C overnight with primary antibodies. After incubation, the slides were extensively washed with PBS, probed with Alexa Fluor 555 goat anti-rabbit IgG or Alexa Fluor 488 goat anti-mouse IgG (Thermo Fisher Scientific, 1:250) and 4′,6-diamidino-2-phenylindole dihydrochloride (DAPI) (Merck Life Science, 1:1000). Images were obtained using the Cytation 3 Cell Imaging Multimode reader (BioTek, AHSI, Milan Italy).

### RNA-seq pipeline

Total RNA was extracted using RNeasy mini kit according to manufacturer’s instructions (Qiagen, Bioset s.r.l., Catanzaro, Italy). RNA integrity for library preparation was determined by analysis of extracted total RNA using a 2100 Bioanalyzer (Agilent Technologies) with RNA 6000 NanoChip. RNA concentrations were measured using Qubit RNA Assay Kit. Libraries were prepared from total RNA according to manufacturer instructions with Illumina Stranded mRNA Prep kit. Libraries quality was evaluated by size analysis on 2100 Bioanlyzer (Chip DNA HS) and concentrations were determined using Qubit DNA HS assay kit (Thermo Fisher Scientific). Sequencing was performed on Illumina Novaseq X plus in the 150PE format. Reads preprocessing was performed by using fastp v0.20.0 [34] applying specific parameters to remove residual adapter sequences and to keep only high-quality data (qualified_quality_phred = 20, unqualified_percent_limit = 30, average_qual = 25, low_complexity_filter = True, complexity_threshold = 30). The percentage of uniquely mapped reads resulted high with the mean value of 89% (mean value for sample: unmapped reads 6%, quality base > q30 90%). Then, passing filter reads were mapped to the genome reference (Homo sapiens) using STAR v2.7.0 [35] with standard parameters, except for sjdbOverhang option set on read length. Genome and transcript annotations provided as input were downloaded from v105 of the Ensembl repository. Alignments were then elaborated by RSEM v1.3.3 [36], to estimate transcript and gene abundances. Subsequently, the sample-specific gene-level abundances were merged into a single raw expression matrix by applying a dedicated RSEM command (rsem-generate-data-matrix). Genes with at least 10 counts in N samples were then selected, where N corresponds to the sample number in the smallest experimental group. Differential expression was computed by edgeR [37] from raw counts in each comparison. Multiple testing controlling procedure was applied and genes with an FDR ≤ 0.1 and logFC > |0.5| were considered differentially expressed. Annotation of differentially expressed genes was performed using the bioMart package [38] into R 4.3, querying available Ensembl Gene IDs and retrieving Gene Names and Entrez gene IDs.

### Data source and differential expression analysis

In silico studies on breast cancer patients were performed using The Cancer Genome Atlas (TCGA) Invasive Breast Cancer Cohort [39], the Molecular Taxonomy of Breast Cancer International Consortium (METABRIC) dataset [40] and the AFFYMETRIX dataset [41], whereas the TCGA Prostate Adenocarcinoma Cohort [42] and the GSE54460 [43] and GSE70770 [44] datasets were used for prostate cancer investigations. mRNA expression data (RNA Seq V2 RSEM) and associated clinical information reported in the Invasive Breast Cancer Cohort of the TCGA project were retrieved from UCSC Xena (https://xenabrowser.net/), samples (n. 1247) were filtered for missing values and by “sample type” to separate tumor tissues (n. 1104) from the adjacent normal tissues (n. 113). The clinical information and the microarray gene expression data (Log_2_ transformed intensity values) of the METABRIC cohort (n. 2509) were downloaded from cBioPortal for Cancer Genomics (http://www.cbioportal.org/). The gene expression levels and clinical information of the AFFYMETRIX cohort (n. 2999) were retrieved from 17 integrated Affymetrix gene expression datasets, as previously described [41]. In brief, the Raw.cel files from 17 AFFYMETRIX U133A/ plus two gene expression datasets of primary breast tumors were retrieved from NCBI Gene Expression Omnibus (GEO: GSE12276, GSE21653, GSE3744, GSE5460, GSE2109, GSE1561, GSE17907, GSE2990, GSE7390, GSE11121, GSE16716, GSE2034, GSE1456, GSE6532, GSE3494), summarized with Ensembl alternative CDF, and then normalized with RMA, before their integration using ComBat to eliminate dataset-specific bias [41]. mRNA expression data (RNA Seq V2 RSEM) and associated clinical information reported in the Prostate Adenocarcinoma cohort of the TCGA project were retrieved from UCSC Xena (https://xenabrowser.net/), samples (n. 550) were filtered for missing values and by “sample type” to separate tumor tissues (n. 498) from the adjacent normal tissues (n. 52). The GSE54460 and GSE70770 datasets were retrieved from NCBI GEO. In order to obtain the differentially expressed genes (DEGs) between breast and prostate carcinomas we used the Breast Invasive Carcinoma and Prostate Adenocarcinoma cohorts of the TCGA PanCancer Atlas [45]. DEGs were calculated using the limma R package considering *p* < 0.05 and log2FC ≥ 1 or log2FC ≤ -1, as thresholds. Heatmaps were drawn with the pheatmap package in R Studio.

### Pathway enrichment analysis

To explore the biological significance of the DEGs obtained from the preceding analysis performed comparing the transcriptome of breast and prostate carcinomas, the *enrichKEGG()* function of the clusterProfiler package [46] was employed in R to assess pathway enrichment analysis. The following parameters were used: organism = “human”, p-value cut-off = 0.05.

### Clustering analysis

A clustering task was executed to partition the patients into several clusters, based on their gene characteristics and similarities. More specifically, the goal is to find groups of patients as homogenous as possible, such that intra-cluster distances (i.e., distances among patients belonging to the same cluster) are minimized, and inter-cluster distances (i.e., distances among patients belonging to different clusters) are maximized. The clustering task has been performed by applying K-means, a classic partitional clustering algorithm that detects centroid-based clusters, where clusters are formed by minimizing the sum of (squared) distances between the points and their respective cluster centroid. In our implementation, we exploited the *kmeans()* function available in the R Stats package. In addition, since the range of gene expression values in the patient samples could vary widely, a min-max normalization task has been performed to rescale the range of gene’s expression values in [0, 1]. Then, the number of clusters *K* has been chosen by adopting a parameter-sweeping methodology; more in detail, the K value maximizing the silhouette (i.e., a clustering quality measure) of the final clustering model has been selected. We exploited also the elbow heuristic, to evaluate the cut-off point of the sum of square error (SSE) curve. In our test, the best clustering quality has been achieved by fixing K = 2. The final result consists of a labeled patient dataset, where a label value (cluster 1 or 2) is associated to each patient.

### Classification analysis

Given the labeled data resulted from the clustering process, a classification task was executed to discover a knowledge model from such data. The goal is to discover a data-driven classifier to be exploited both as descriptive model (i.e., which genes and/or gene value ranges affect class values) and predictive model (i.e., assign a class value to a new previously unseen patient, as accurately as possible). The classification task has been performed by learning decision tree models, which are some of the most important and representative classification models adopted in the field of machine learning. In our faced scenario, the hierarchical tree-based model is built, node by node (from the root to the leaves), by selecting the best gene that splits the patients into the two predefined categories. In particular, at each node, it is chosen the splitting gene providing the highest information gain (or the highest reduction in entropy) in the data. The resulting tree can be also represented in a set of decision rules, which can be used to understand which gene affects the patient partitions, and/or to make predictions or classify new patients. To perform the decision tree learning and its validation, the original patient dataset was split in two partitions: the training set and the test set, in the ratio of 70% and 30%, respectively. The classification trees are learned from the training set, then the trained models are exploited to make predictions on the test set, to assess the predictive effectiveness of the approach. Furthermore, to have a statistically robust estimation of classification model performances, we run our tests by implementing the k-fold cross-validation methodology (k = 25, in our case), which is a resampling method executing k train-test iterations on different portions of the data. Classification performance has been assessed both by computing accuracy, precision, and recall measures, and by building the classification matrix for each specific test set.

### Survival analysis

The survival analyses on breast cancer patients were performed using the TCGA, METABRIC, and AFFYMETRIX gene expression data along with the overall survival information. Patients of the METABRIC cohort classified as “died of other causes” were excluded from the analysis. Survival analyses on prostate cancer patients were performed using the TCGA gene expression data and the disease-free interval and progression-free interval; cumulative gene expression information of the GSE54460 [43] and GSE70770 [44] datasets were employed to predict the biochemical recurrence. The Kaplan-Meier survival curves were generated using the survival and the survminer R packages. A log-rank test was used to determine differences between the survival curves. *p* < 0.05 was considered statistically significant.

## Results

### Transcriptional landscape of breast and prostate CAFs

**Fig. 1:**
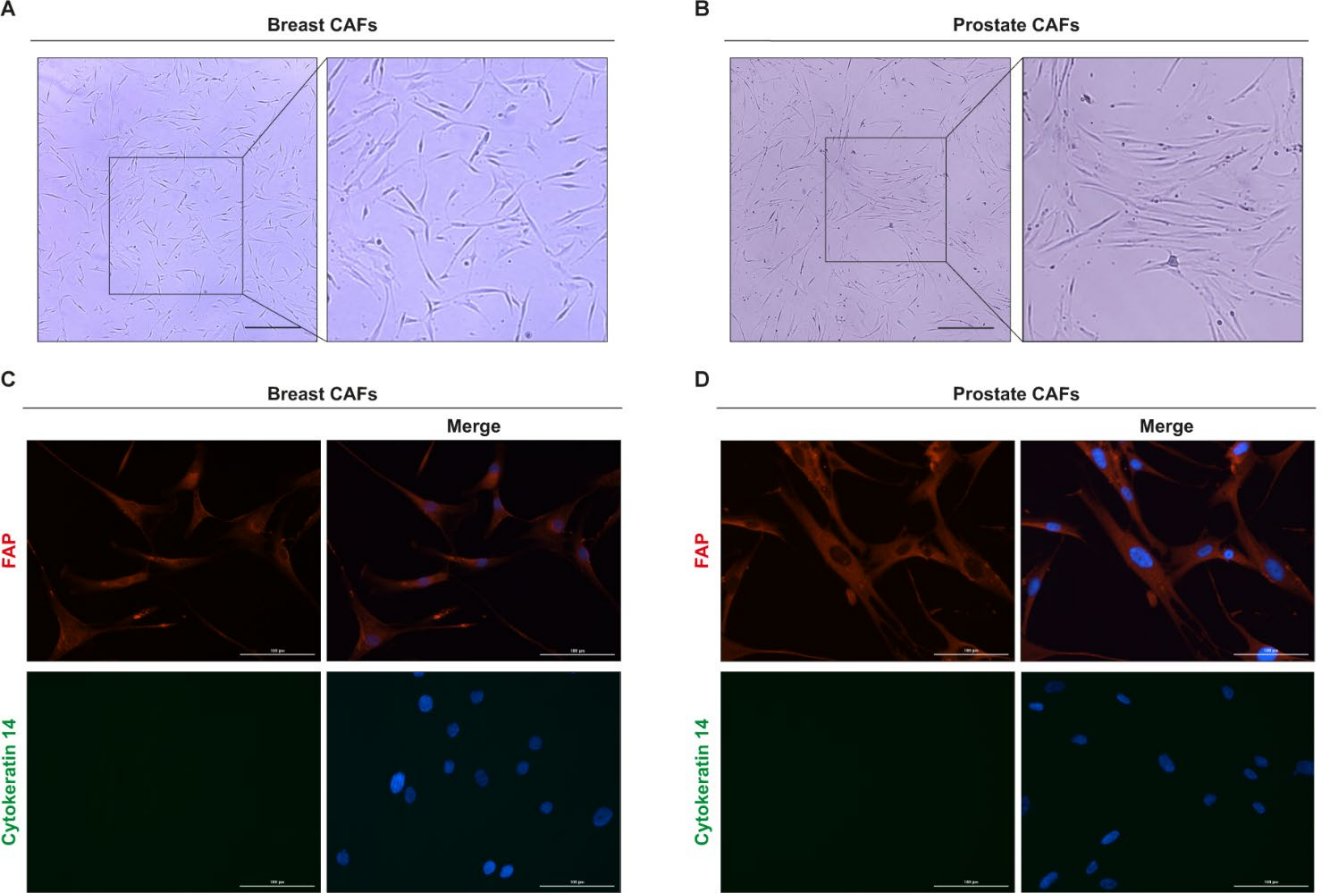
Characterization of breast and prostate CAFs. Morphological appearance of breast (**A**) and prostate (**B**) CAFs observed by phase-contrast microscopy; scale bar: 650 μm. Enlarged details are shown in the side boxes. FAP (red signal) and Cytokeratin 14 (green signal) immunofluorescence staining in breast (**C**) and prostate (**D**) CAFs. Nuclei were stained by DAPI (blue signal). Scale bar: 100 μm. The images shown represent 10 random fields from three independent experiments

**Fig. 2:**
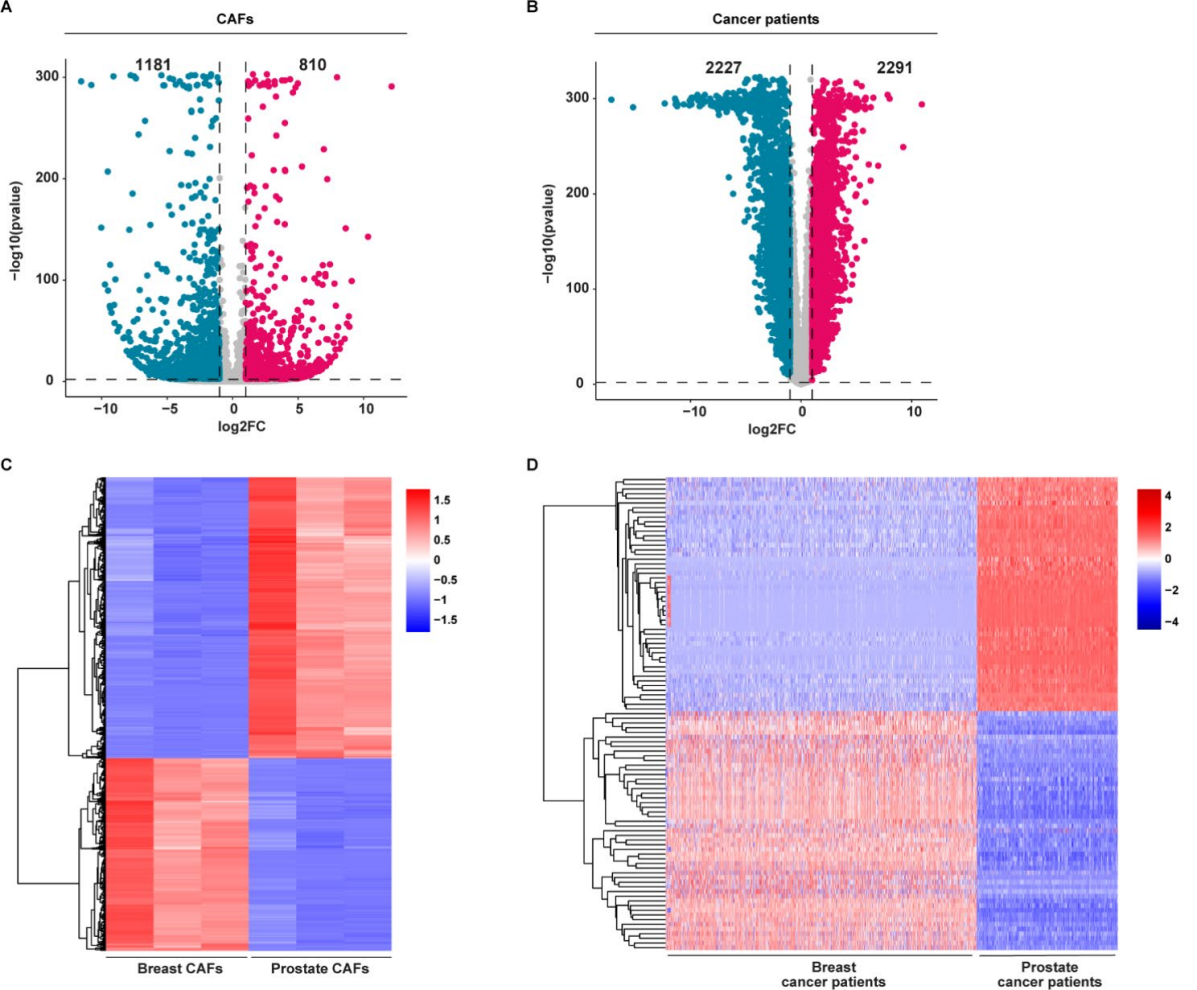
Identification of the DEGs in breast and prostate CAFs and primary tumors. Venn diagram (**A**), and heat map (**C**) showing the DEGs in breast and prostate CAFs, as ascertained by RNA-sequencing analysis (log2FC ≥ 1 or log2FC ≤ -1; *p* ≤ 0.01). Venn diagram (**B**), and heat map (**D**) showing the DEGs in breast and prostate cancer patients of the TCGA dataset (log2FC ≥ 1 or log2FC ≤ -1; *p* ≤ 0.01). In the volcano plots, significantly down-regulated genes are shown in blue, significantly up-regulated genes are shown in pink, non-significant genes are shown in grey (*p* > 0.01)

Considering that CAFs are a highly heterogeneous cellular population within the TME characterized by a context-dependent influence on cancer progression [27, 47], in the present study we aimed to assess the transcriptional profile of both breast and prostate CAFs. First, CAFs isolated from surgically resected breast and prostate carcinomas (Fig. 1A-B) and characterized by immunofluorescent staining, revealed the expression of FAP and the absence of the epithelial marker cytokeratin 14 (Fig. 1C-D). Then, RNA-seq analysis was performed to comprehensively delineate the gene expression patterns of CAFs derived from breast and prostate tumors. Of note, 810 genes were found up-regulated (log2FC ≥ 1, *p* ≤ 0.01) and 1181 genes were found down-regulated (log2FC ≤ -1, *p* ≤ 0.01) in breast with respect to prostate CAFs (Fig. 2A, C). Subsequently, in order to strengthen the results obtained from our in vitro models, we took advantage of the breast invasive carcinoma and prostate adenocarcinoma cohorts of the TCGA PanCancer Atlas [45]. By calculating the DEGs in the breast with respect to prostate cancer patients, we found 2291 up-regulated genes (log2FC ≥ 1, *p* ≤ 0.01) and 2227 down-regulated genes (log2FC ≤ -1, *p* ≤ 0.01) in breast with respect to prostate cancer patients (Fig. 2B, D). It should be pointed out that the genes up-regulated in breast vs. prostate CAFs or in cancer patients are here indicated as down-regulated in prostate vs. breast CAFs or in cancer patients, while the genes down-regulated in breast vs. prostate CAFs or cancer patients are here indicated as up-regulated in prostate vs. breast CAFs or cancer patients.

### Clustering analysis reveals a CAFs-derived gene signature associated with poor prognosis in breast cancer patients

**Fig. 3:**
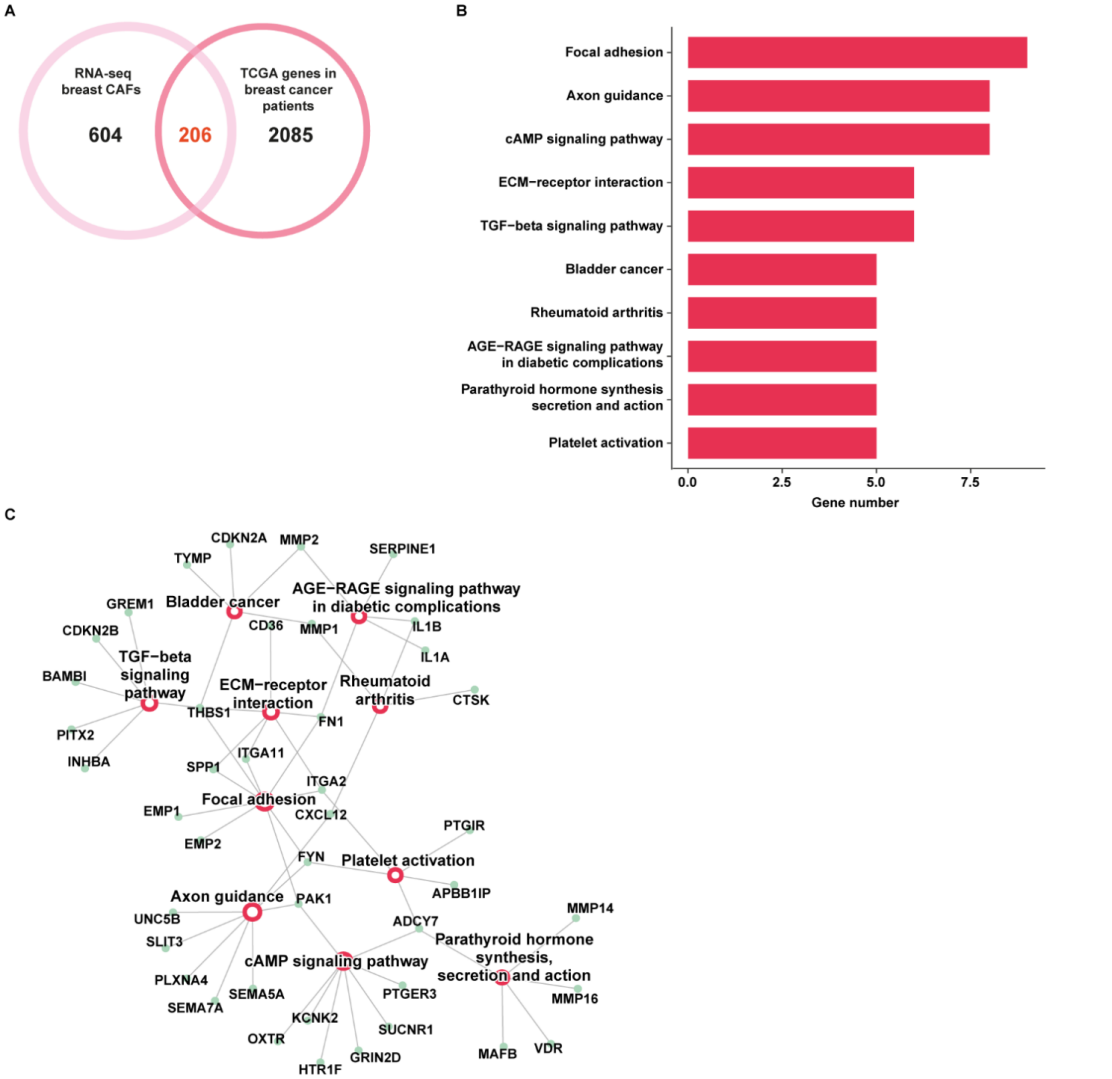
Pathway enrichment analysis of the genes up-regulated in breast CAFs and cancer patients. (**A**) Venn diagram showing the intersection of the genes up-regulated in breast CAFs and cancer patients of the TCGA cohort. (**B**) KEGG pathway analysis of the 206 genes commonly up-regulated in breast CAFs and breast cancer samples of the TCGA dataset. The number of genes in the identified pathways is displayed along the X-axis, while the different KEGG terms are shown along the Y-axis (*p* < 0.05). (C) Connection plot showing the interrelation among the KEGG pathways and genes

**Fig. 4:**
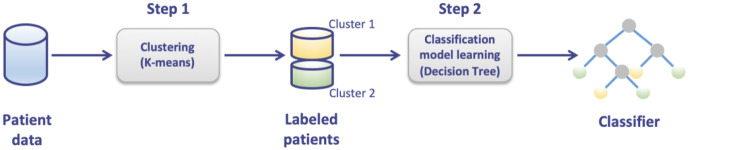
Workflow providing a graphic overview of the clustering and classification analyses. In step 1, a clustering task is executed to partition the patients into two groups (or clusters), based on the gene expression similarities of the patients. The result of this step consists of a labeled patient dataset, where a label value (cluster 1 or 2) is associated with each patient. In step 2, given the labeled data resulted from the clustering process, a classification task was executed to discover a decision tree classifier from such data. The detected classifier can be exploited as either a descriptive model (i.e., understanding which genes and/or gene value ranges affect class values) or a predictive model (i.e., assigning a class value to a new previously unseen patient)

We focused our attention on the genes up-regulated either in breast CAFs or in breast cancer patients to bridge in vitro findings to clinical implications. In this regard, by intersecting the 810 up-regulated genes in breast CAFs with the 2291 up-regulated genes in breast cancer patients of the TCGA dataset, we found 206 common genes (Fig. 3A). In order to investigate the biological significance of these genes, we performed KEGG (The Kyoto Encyclopedia of Genes and Genomes) pathway analysis. The aforementioned genes were enriched in several pathways, as schematically shown in Fig. 3B-C. We focused our attention on the “Focal adhesion” pathway since it comprises the highest number of genes (*ITGA11*, *THBS1*, *FN1*, *EMP1*, *ITGA2*, *FYN*, *SPP1*, *EMP2*, and *PAK1*) and correlates with aggressive cancer features [48]. To uncover whether these genes may be implicated in the stratification of breast cancer patients, we performed an unsupervised clustering analysis using k-means followed by classification using a decision tree algorithm (Fig. 4).

**Fig. 5:**
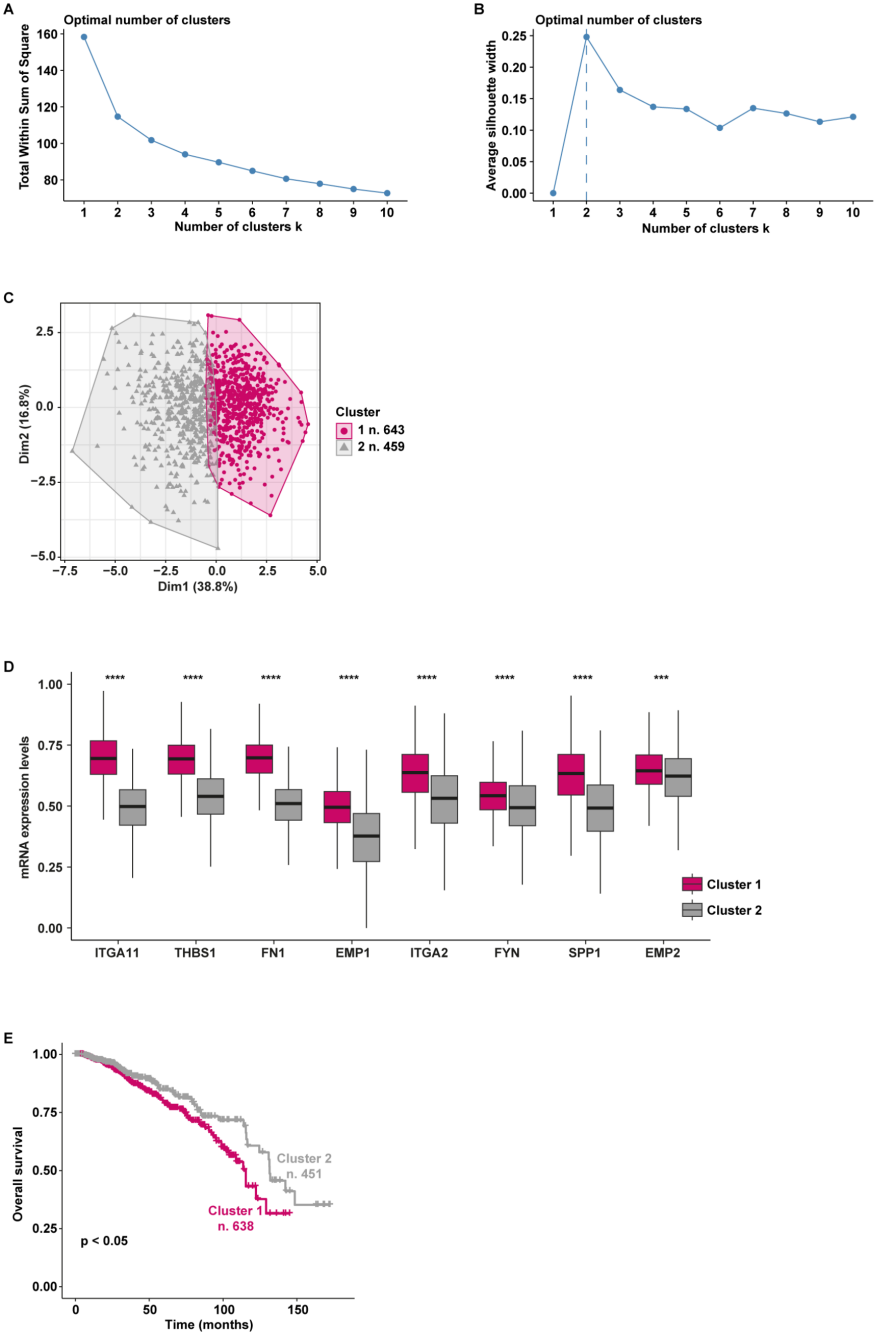
Survival analysis of TCGA breast cancer patients clustered based on the levels of the focal adhesion pathway’s genes. The optimal number of k-means clusters was determined and visualized using within-cluster sums of squares (**A**) and average silhouette (**B**) methods. (**C**) Visualization of k-means partitioning method; observations (breast cancer patients of the TCGA dataset) are represented by points in the plot, using principal components. Each data point in the reduced-dimensional space is color-coded based on its assigned cluster, the number of patients belonging to each cluster is shown. (**D**) Multiple boxplots showing the differential expression of the 8 genes in the two clusters. (**E**) Overall survival of breast cancer patients belonging to cluster 1 and cluster 2. (***) indicates *p* < 0.001; (****) indicates *p* < 0.0001

**Fig. 6:**
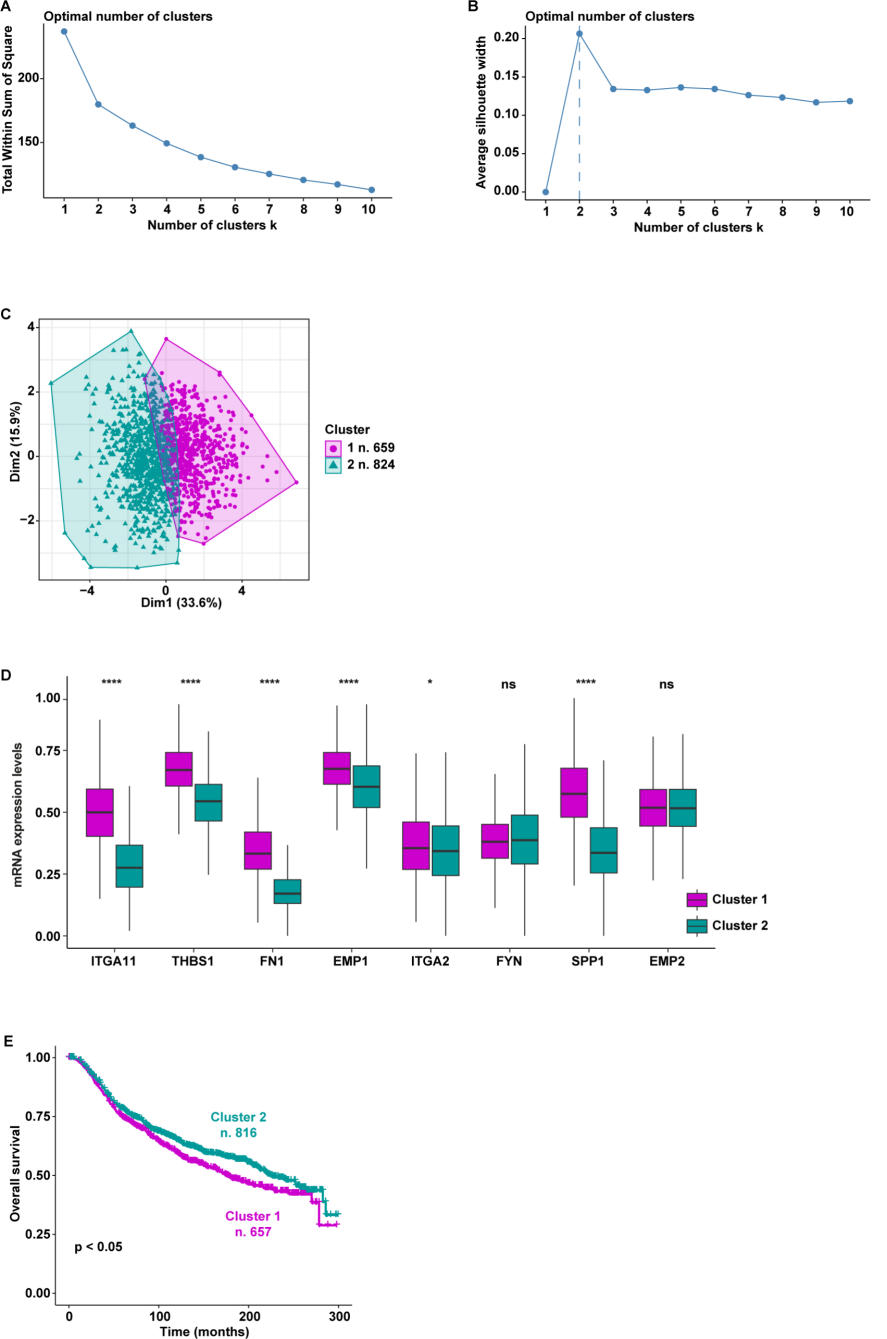
Survival analysis of METABRIC breast cancer patients clustered based on the levels of the focal adhesion pathway’s genes. The optimal number of k-means clusters was determined and visualized using within-cluster sums of squares (**A**) and average silhouette (**B**) methods. (**C**) Visualization of k-means partitioning method; observations (breast cancer patients of the METABRIC dataset) are represented by points in the plot, using principal components. Each data point in the reduced-dimensional space is color-coded based on its assigned cluster, the number of patients belonging to each cluster is shown. (**D**) Multiple boxplots showing the differential expression of the 8 genes in the two clusters. (**E**) Overall survival of breast cancer patients belonging to cluster 1 and cluster 2.; ns indicates non-significant; (*) indicates *p* < 0.05; (****) indicates *p* < 0.0001

**Fig. 7:**
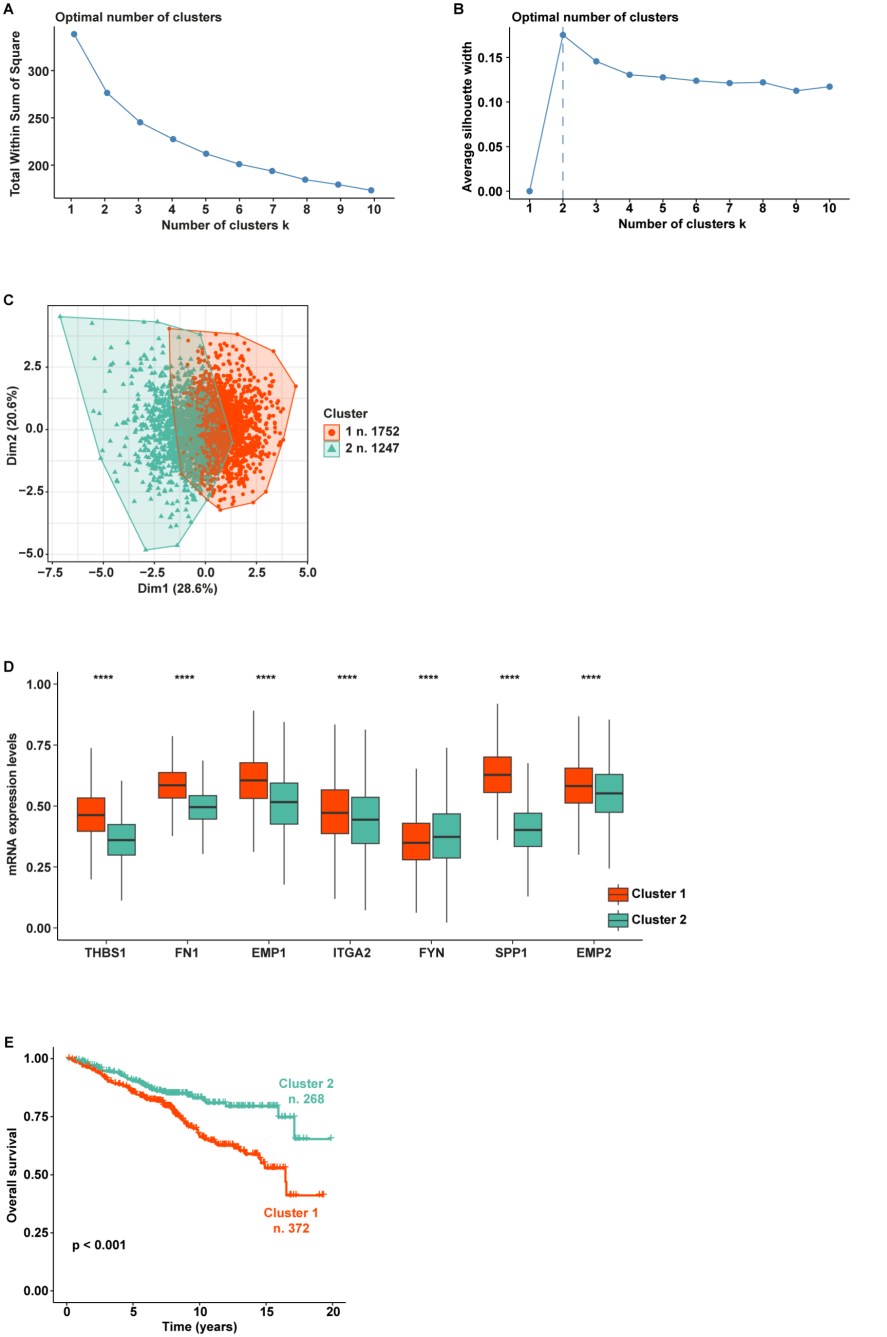
Survival analysis of AFFYMETRIX breast cancer patients clustered based on the levels of the focal adhesion pathway’s genes. The optimal number of k-means clusters was determined and visualized using within-cluster sums of squares (**A**) and average silhouette (**B**) methods. (**C**) Visualization of k-means partitioning method; observations (breast cancer patients of the AFFYMETRIX dataset) are represented by points in the plot, using principal components. Each data point in the reduced-dimensional space is color-coded based on its assigned cluster, the number of patients belonging to each cluster is shown. (**D**) Multiple boxplots showing the differential expression of the 8 genes in the two clusters. (**E**) Overall survival of breast cancer patients belonging to cluster 1 and cluster 2. (****) indicates *p* < 0.0001

In particular, on the basis of the expression levels of the genes belonging to the “Focal adhesion” pathway, TCGA breast cancer patients were assigned to two different clusters according to the optimal number of clusters, which was calculated by the within-cluster sums of squares and average silhouette methods (Fig. 5A-C). Worthy, the patients belonging to cluster 1 (n. 643) displayed a higher expression of 8 out of 9 genes enriched in the “Focal adhesion” pathway (*PAK1* was excluded in the first analysis as it was not differentially expressed between the 2 clusters, thus resulting as a gene not introducing relevant differences between patients belonging to different classes) respect to the patients of cluster 2 (n. 459) (Fig. 5D). Aiming to uncover a clinical relevance of the two clusters of patients, we performed survival analysis showing that breast cancer patients characterized by high levels of *ITGA11*, *THBS1*, *FN1*, *EMP1*, *ITGA2*, *FYN*, *SPP1*, and *EMP2* (cluster 1) display a worse overall survival compared to the patients showing low expression of the aforementioned genes (cluster 2) (Fig. 5E). Based on these data suggesting that the 8 genes identified may predict aggressive features of breast tumors, we aimed to corroborate our findings by using further cohorts of breast cancer patients. In this regard, the same workflow was applied to breast cancer patients of the METABRIC and AFFYMETRIX datasets. In accordance with the within-cluster sums of squares and average silhouette methods, the patients of the METABRIC cohort were clustered into 2 groups (Fig. 6A-C). Patients belonging to cluster 1 (n. 659) displayed higher expression levels of 6 out of 8 genes with respect to patients of cluster 2 (n. 824) (Fig. 6D). *FYN* and *EMP2* were not differentially expressed between the two METABRIC clusters. Importantly, survival analysis revealed that breast cancer patients of the METABRIC cohort belonging to cluster 1 exhibit a significantly worse overall survival when compared to patients of cluster 2 (Fig. 6E), consistent with the findings obtained on TCGA breast cancer patients. Remarkably, this approach unveiled 2 distinct clusters within the AFFYMETRIX cohort of breast cancer patients. In particular, we found 2 clusters of patients based on the expression of the identified genes (with the exception of *ITGA11* which is not annotated in the AFFYMETRIX dataset) (Fig. 7A-C). In particular, patients of cluster 1 (n. 1752) showed higher expression levels of *THBS1*, *FN1*, *EMP1*, *ITGA2*, *SPP1*, and *EMP2* compared to patients of cluster 2 (n. 1247). Notably, patients characterized by a high expression of the aforementioned genes (cluster 1) display worse overall survival with respect to patients showing low gene levels (cluster 2), as indicated in Fig. 7E.

**Fig. 8:**
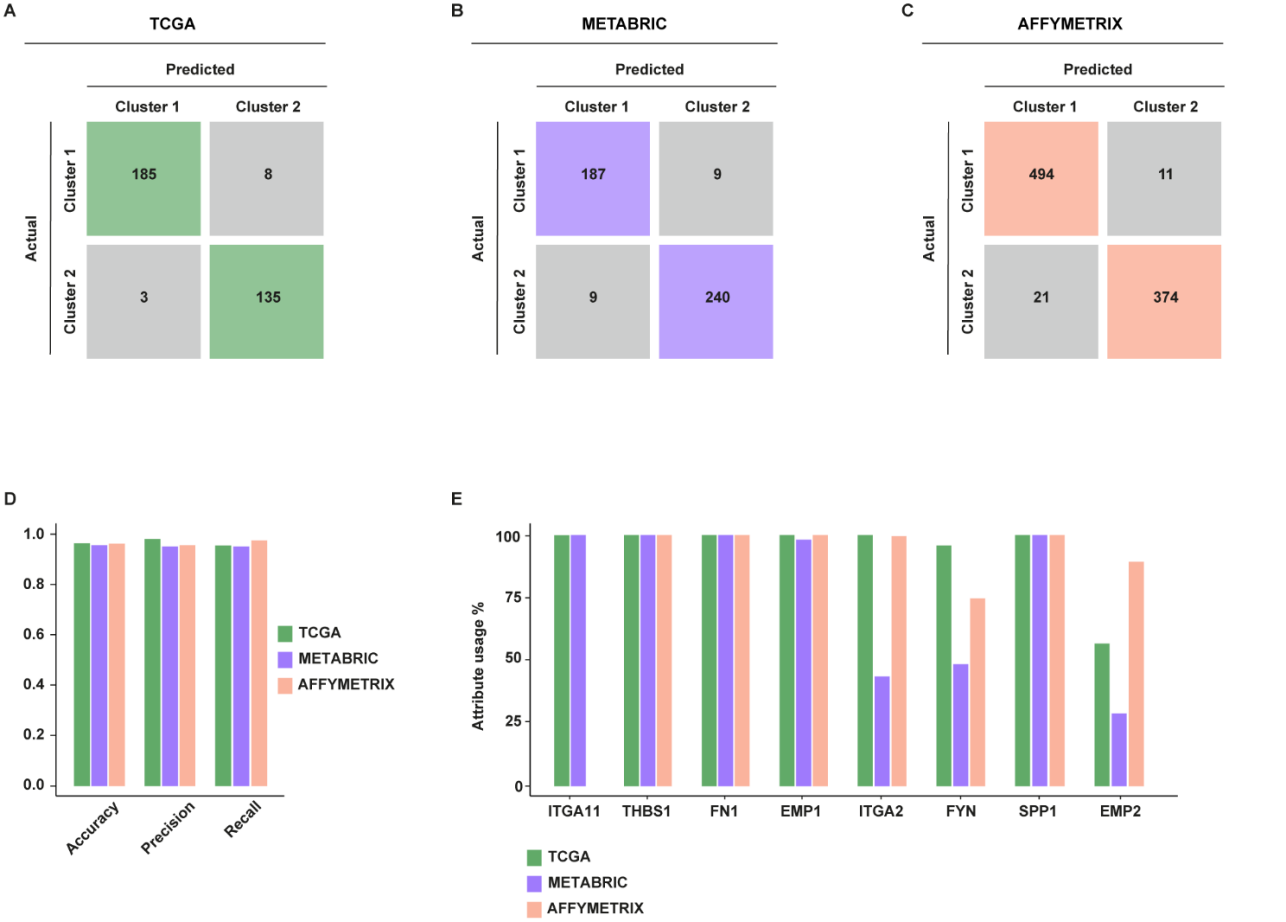
Validation of k-means clustering of breast cancer patients through decision tree classification analysis. Confusion matrixes provide quantitative evaluations of the model’s performance on the TCGA (**A**), METABRIC (**B**), and AFFYMETRIX (**C**) datasets; matrix rows correspond to the actual classes, columns represent predicted classes, each squared box contains the count of instances falling into a specific category. (**D**) Histogram showing accuracy, recall, and precision of the classification models on the TCGA, METABRIC, and AFFYMETRIX datasets. (**E**) Attribute usage percentage of the 8 genes through the boosting algorithm on the training dataset (25 trials)

Aiming to strengthen the results obtained by the clustering process, we trained a decision tree where the breast CAFs-derived gene signature has been considered as a descriptive feature, and the labels achieved by clustering as class values. The samples of the TCGA, METABRIC, and AFFYMETRIX datasets were split into training and testing sets, and the performance evaluation was carried out by computing accuracy, precision, and recall, as described in the Methods section. Finally, the confusion matrix for each dataset has been built (Fig. 8A-C). Of note, the breast CAFs-related gene signature yielded 0.967 accuracy, 0.984 precision, and 0.958 recall in the TCGA dataset, 0.959 accuracy, 0.954 precision, and 0.954 recall in the METABRIC dataset, 0.965 accuracy, 0.959 precision, and 0.978 recall in the AFFYMETRIX dataset (Fig. 8D). The attribute usage calculated by the voting of the boosted algorithm on the training set is shown in Fig. 8E, showing that *ITGA11*, *THBS1*, *FN1*, *EMP1*, and *SPP1* result the most appropriate genes discriminating between the two categories. Together, these results indicate that poor survival rates characterize breast cancer patients showing high expression of the identified gene signature, which may be therefore useful to predict the outcome of breast tumor patients.

### Identification of a CAFs-derived gene signature with prognostic value in prostate cancer patients

**Fig. 9:**
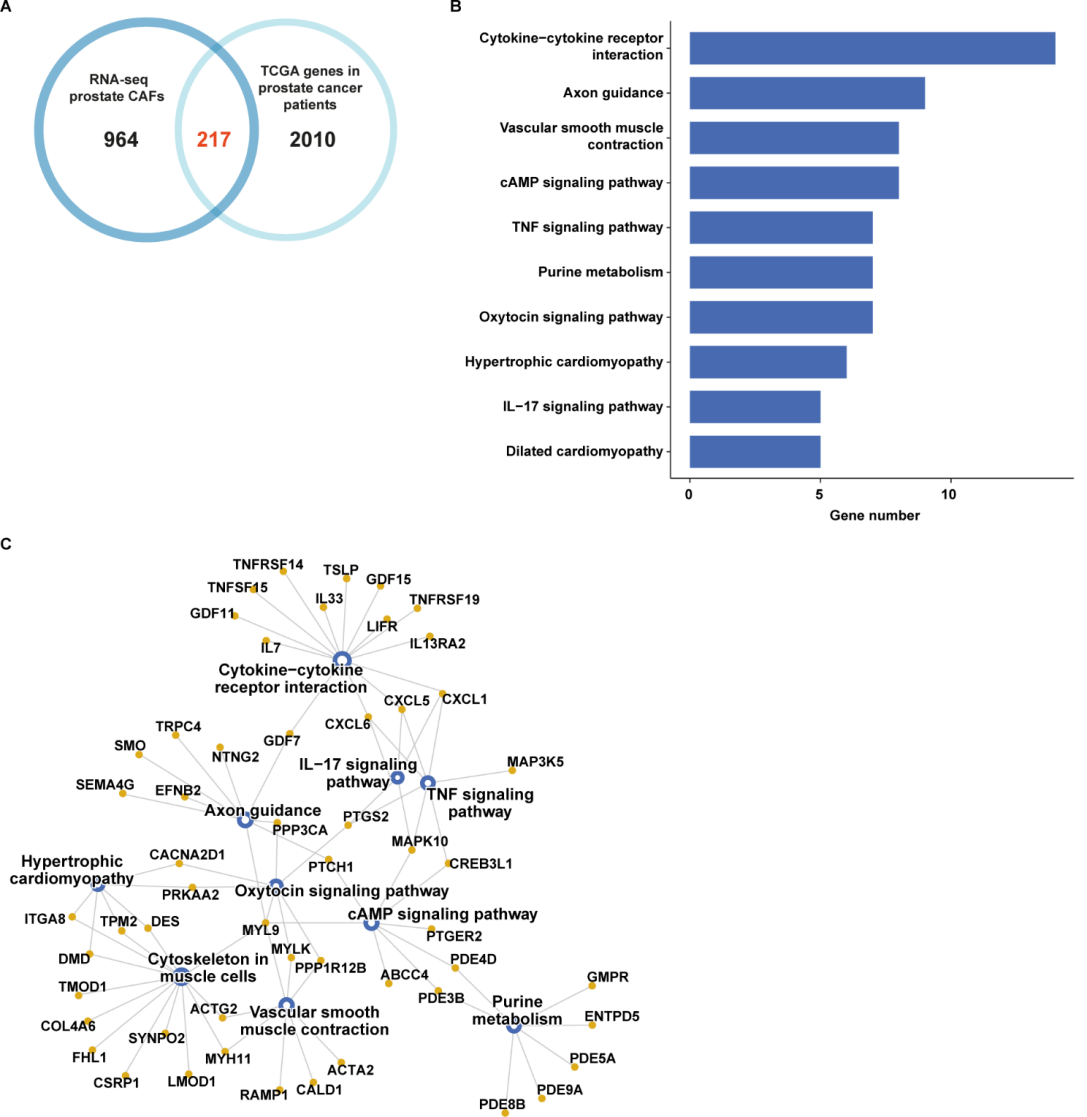
Pathway enrichment analysis of the genes up-regulated in prostate CAFs and cancer patients. (**A**) Venn diagram showing the intersection of the genes up-regulated in prostate CAFs and patients of the TCGA cohort. (**B**) KEGG pathway analysis of the 217 genes commonly up-regulated in prostate CAFs and prostate cancer samples of the TCGA dataset. The number of genes in the identified pathways is displayed along the X-axis, while the different KEGG terms are shown along the Y-axis (*p* < 0.05). (**C**) Connection plot showing the interrelation among the KEGG pathways and genes

**Fig. 10:**
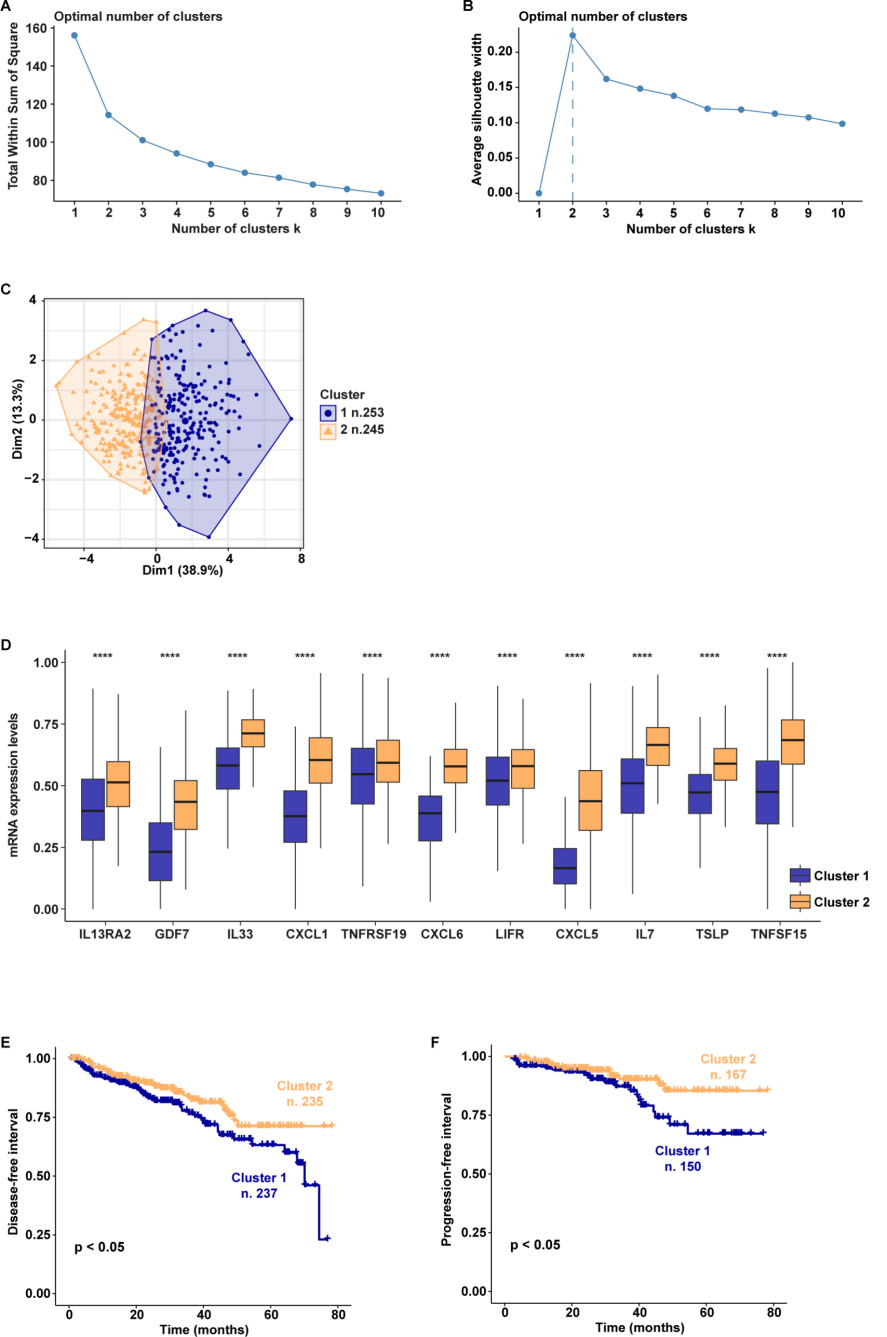
Survival analysis of TCGA prostate cancer patients clustered based on the levels of the genes belonging to the cytokine-cytokine receptor interaction pathway. The optimal number of k-means clusters was determined and visualized using within-cluster sums of squares (**A**) and average silhouette (**B**) methods. (**C**) Visualization of k-means partitioning method; observations (prostate cancer patients of the TCGA dataset) are represented by points in the plot, using principal components. Each data point in the reduced-dimensional space is color-coded based on its assigned cluster, the number of patients belonging to each cluster is shown. (**D**) Multiple boxplots showing the differential expression of the 11 genes in the two clusters. Disease-free interval (**E**) and progression-free interval (**F**) of prostate cancer patients belonging to cluster 1 and cluster 2. (****) indicates *p* < 0.0001

Aiming to ascertain whether the CAFs-derived genes resulting from our RNA-seq analysis may have a clinical implication, we intersected the 1181 up-regulated genes in prostate CAFs with the 2227 up-regulated genes in prostate cancer patients. We obtained 217 joint genes (Fig. 9A), which were found enriched in a number of pathways, as revealed by KEGG pathway analysis (Fig. 9B-C). Notably, the “Cytokine-cytokine receptor interaction” pathway appeared to comprise the highest number of genes (*IL13RA2*, *GDF7*, *IL33*, *CXCL1*, *TNFRSF19*, *CXCL6*, *LIFR*, *CXCL5*, *IL7*, *TSLP*, *TNFSF15*, *GDF11*, *TNFSF14*, and *GDF15*). In order to uncover whether these genes may have a clinical significance, we performed k-means clustering analysis on the prostate cancer patients of the TCGA cohort. The patients were assigned to two different clusters following the optimal number of clusters, which was calculated by the within-cluster sums of squares and average silhouette methods (Fig. 10A-C). Patients belonging to cluster 2 (n. 245) displayed a higher expression of 11 out of 14 genes from the “Cytokine-cytokine receptor interaction” pathway with respect to patients of cluster 1 (n. 253) (Fig. 10D). *GDF11*, *TNFSF14*, and *GDF15* were excluded from the analysis since they were not differentially expressed between the 2 clusters. Interestingly, by performing survival analysis we found that patients belonging to cluster 1 display a poor outcome with respect to patients of cluster 2 in terms of both disease-free interval (Fig. 10E) and progression-free interval (Fig. 10F).

**Fig. 11:**
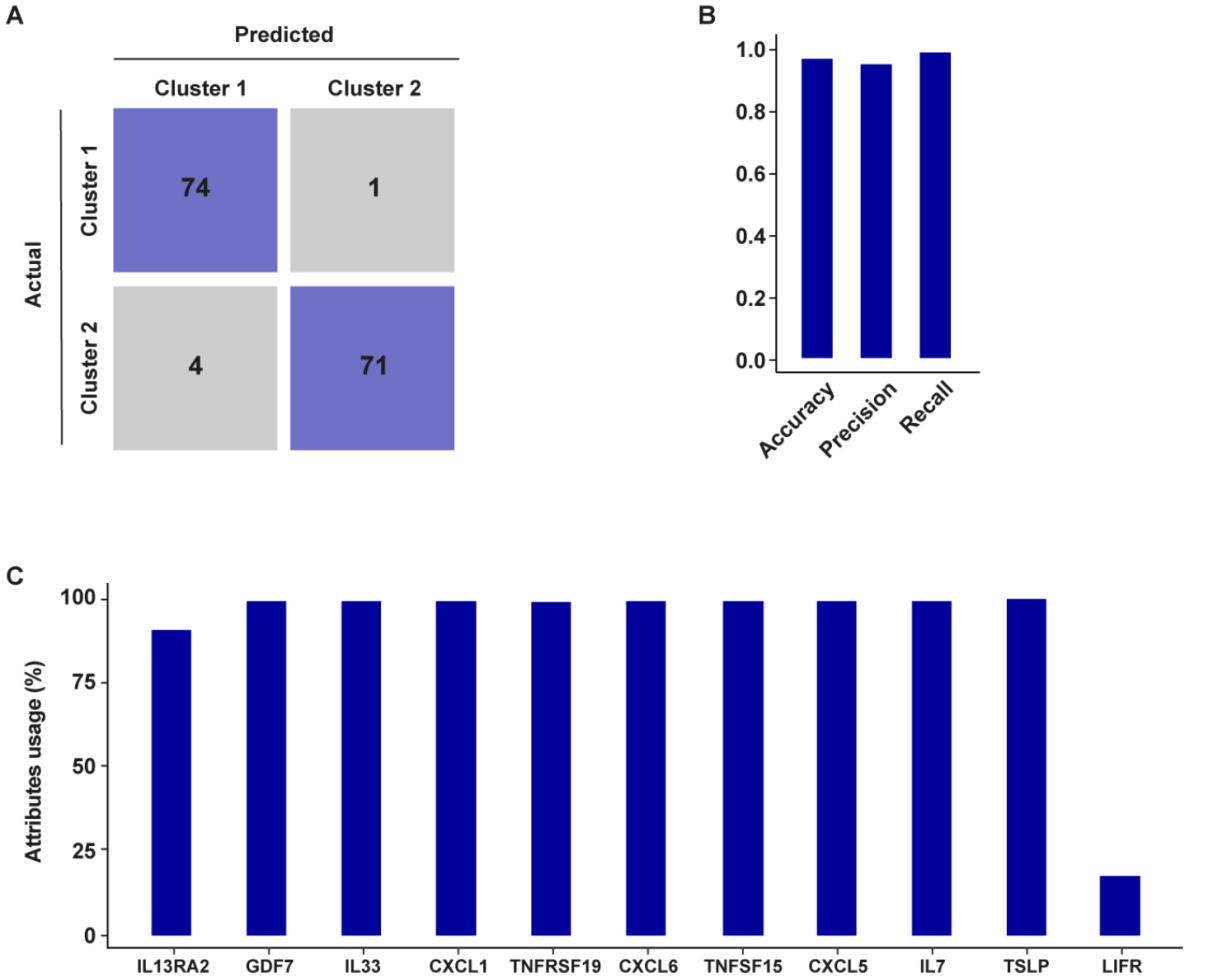
Validation of k-means clustering of prostate cancer patients through decision tree classification analysis. (**A**) The confusion matrix provides a quantitative evaluation of the model’s performance on the TCGA dataset; matrix rows correspond to the actual classes, columns represent predicted classes, each squared box contains the count of instances falling into a specific category. (**B**) Histogram showing accuracy, recall, and precision of the classification model. (**C**) Attribute usage percentage of the 11 genes through the boosting algorithm on the training dataset (25 trials)

As a further step, we conducted a classification analysis to enhance the explainability of the clustering results achieved on the TCGA cohort of prostate cancer patients. We trained a decision tree model, and we tested it on a test set. Then, we assessed the model performance by computing accuracy, precision, and recall, and we built the corresponding confusion matrix (Fig. 11A). Of note, the prostate CAFs-derived gene signature generated 0.966 accuracy, 0.948 precision, and 0.986 recall (Fig. 11B). Figure 11C shows the attribute usage, demonstrating that the patient’s cluster membership is mainly represented by all the evaluated genes except for *LIFR*.

**Fig. 12:**
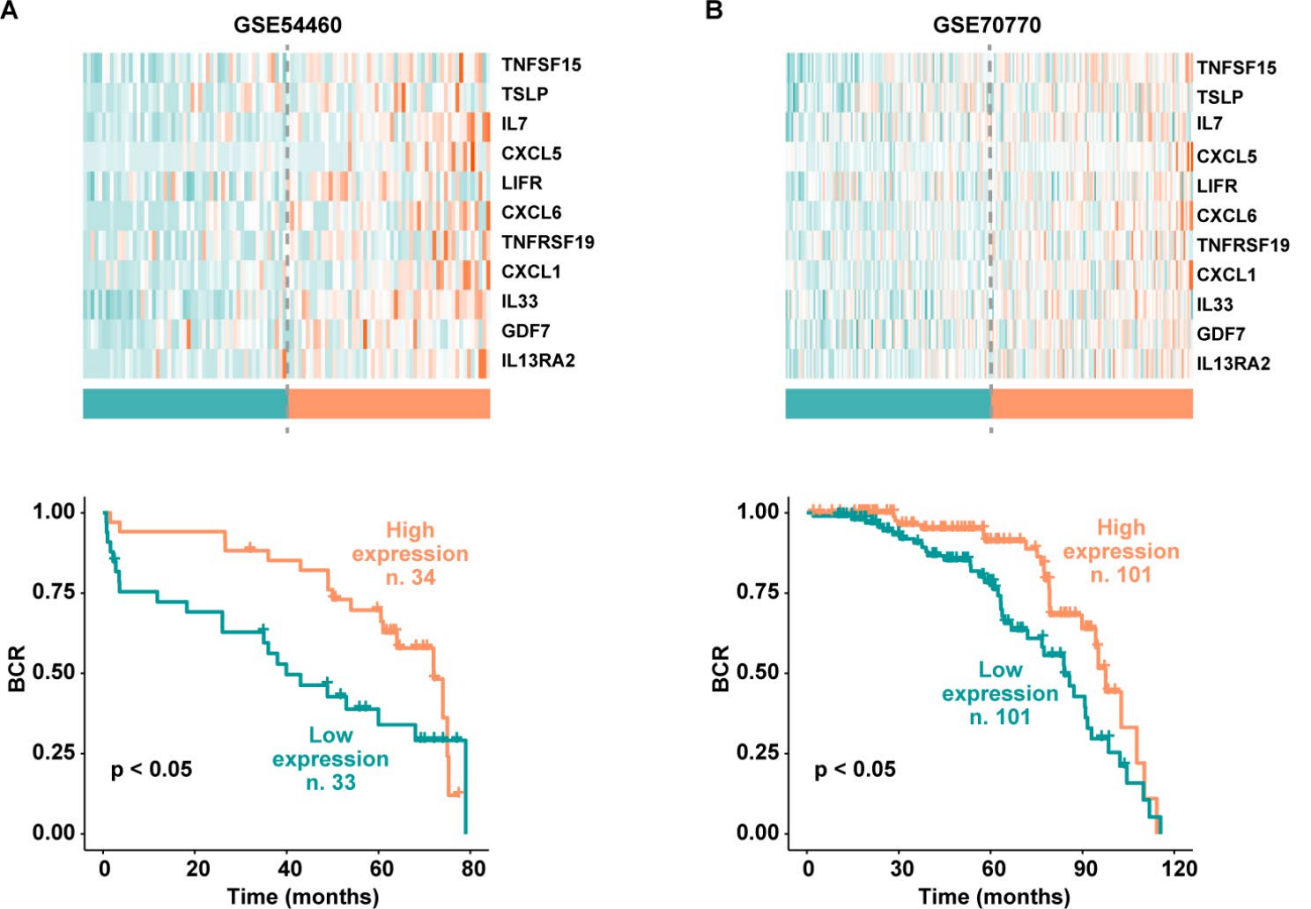
Prostate cancer recurrence based on the cumulative levels of the genes belonging to the cytokine-cytokine receptor interaction pathway. Kaplan-Meier curves showing the association of high or low expression of the genes belonging to the cytokine-cytokine receptor interaction pathway and biochemical recurrence (BCR) in prostate cancer patients of the GSE54460 (**A**) and GSE70770 (**B**) datasets

Thereafter, we evaluated the prognostic role of the 11 genes using two further datasets of prostate cancer patients, namely GSE54460 and GSE70770 [43, 44]. In accordance with the results obtained on the TCGA cohort, survival analysis revealed that prostate cancer patients showing a low cumulative expression of the 11 genes display a poor prognosis (Fig. 12A-B). Overall, these data suggest that the identified CAFs-derived genes may be considered prognostic indicators of positive survival rates in prostate carcinomas.

## Discussion

It is widely accepted that the molecular and biological complexity of a tumor relies on the liaison between cancer cells and the surrounding TME, which is a dynamic ecosystem comprising diverse cell types [49, 50]. Among these stromal cellular components, CAFs are recognized as the most prevalent in the majority of solid tumors, including breast and prostate cancers [23, 24]. Accumulating evidence underscored the heterogeneity of CAFs populations [27, 47, 51]. In particular, CAFs may play a pivotal role in fostering several pro-tumorigenic processes, such as cancer cell migration and invasion, cancer stem-cell renewal, development of chemoresistance, and evasion of immune responses [18, 49]. Nevertheless, in certain tumor contexts, CAFs may exert a suppressive function within the TME [52]. In this intricate scenario, marker genes that allow to differentiate tumor-promoting from suppressive CAFs in diverse types of tumors, remain to be discovered.

In the present study, we have isolated and characterized CAFs from breast and prostate cancer patients. The comparison between their transcriptomic profile by RNA-seq analysis revealed tumor-specific molecular features. The intersection between the DEGs derived from CAFs and those obtained from cancer patients allowed us to identify two tumor context-specific gene signatures. Using large cohorts of breast and prostate cancer patients and machine learning techniques, we have further addressed the valuable predictive utility of the two CAFs-related gene signatures and provided insights into the potential of CAFs to drive diverse signaling and biological events in distinct cancer types.

First, we have developed and validated a breast CAFs-derived gene signature based on the transcriptomic profiles of breast cancer patients from 3 datasets. Worthy, the signature demonstrated to predict poor outcomes in breast cancer patients exhibiting high transcriptional levels of *ITGA11*, *THBS1*, *FN1*, *EMP1*, *ITGA2*, *FYN*, *SPP1*, and *EMP2*.

In accordance with our data, the pro-tumorigenic role and the poor prognostic value of the “Focal adhesion” pathway, which comprises the genes belonging to the breast CAFs-derived signature, have been largely recognized [48]. Accumulated evidence indicates that the focal adhesion signaling functions as a signaling hub in ECM-tumor interactions as well as breast cancer cell adhesion, survival, proliferation, migration, and invasion [53, 54]. The core component of the focal adhesion axis is the focal adhesion kinase (FAK) [55]. Upon activation by integrins, FAK can establish complexes with diverse intracellular molecules to allow the bidirectional transmission of mechanical and biochemical signals across the plasma membrane, therefore regulating a variety of stimulatory responses in cancer cells [55, 56]. In addition, the pro-tumorigenic role of FAK is further supported by its ability to regulate the transcription of pro-inflammatory molecules that suppress destructive host immunity, thus promoting a favorable TME [57]. Moreover, FAK signaling has been shown to support pro-metastatic TME remodeling by regulating angiogenesis, vascular permeability, and ECM production [57]. Among the genes belonging to the FAK signaling, integrins are cell adhesion receptors for ECM molecules influencing the potential of cancer cells to grow as well as escape from the primary tumor, invade, survive in the blood circulation, and metastasize to distant sites [58, 59]. In addition, integrins may serve as main mediators of the crosstalk between cancer cells and TME components like CAFs [60, 61]. In this scenario and according to our findings, α11 integrin (*ITGA11*) is significantly overexpressed in the breast stromal compartment and linked to aggressive tumor features, such as high histologic grade and proliferative rate [62]. Known ligands for certain integrin receptors are the fibronectin 1 (*FN1*) and the secreted phosphoprotein 1 (*SPP1*, also known as osteopontin). The glycoprotein fibronectin 1 serves as a hallmark of CAFs and has been indicated as a facilitator of breast tumor stroma remodeling, for instance by promoting CAFs-mediated migration and invasion of tumor cells [63, 64]. As it concerns osteopontin, its secretion by CAFs enhances breast tumor growth [65, 66], while cancer-derived osteopontin plays a role in reprograming normal fibroblasts into tumor-promoting CAFs [67]. Thrombospondin-1 (*THBS1*) is a matricellular ECM protein that can promote breast tumor migration and invasiveness via the activation of diverse signaling pathways including FAK signaling [68]. Worthy, in CAFs the expression levels of thrombospondin-1 along with other ECM components are correlated with an increased odd ratio for lymph node metastasis [69]. In addition, evidence indicates the association of further cell surface proteins named epithelial membrane proteins (EMPs) with cancer progression and metastasis, even though the precise function of each family member remains to be fully clarified [70]. For instance, the epithelial membrane protein 1 (*EMP1*) is highly expressed in patients with invasive lobular breast tumors, which show a lower survival rate than ductal [71, 72]. However, reduced protein levels of the epithelial membrane protein 1 have been correlated with poor prognosis, and its overexpression in breast cancer cells inhibits their proliferative, and invasive behavior [70]. On the contrary, the pro-metastatic role of the epithelial membrane protein 2 (*EMP2*) in breast cancer is well recognized. It is overexpressed in invasive breast tumors, particularly in the triple-negative molecular subtype, and in lymph node metastases [73].

Next, by taking advantage of the analysis of transcriptome data of both CAFs and TCGA cohorts of prostate cancer patients, we defined a CAFs-derived signature with an unfavorable prognostic value in prostate tumor patients. Functional pathways enrichment analysis revealed that these CAFs-related genes (*IL13RA2*, *GDF7*, *IL33*, *CXCL1*, *TNFRSF19*, *CXCL6*, *LIFR*, *CXCL5*, *IL7*, *TSLP*, and *TNFSF15*) are significantly enriched within the “Cytokine-cytokine receptor interaction” pathway. Even though several reports have indicated that many cytokines, their receptors, and cytokine signaling effectors are engaged in the metastatic process [74], our findings revealed that prostate cancer patients, which were specifically clustered for a low cumulative expression of *IL13RA2*, *GDF7*, *IL33*, *CXCL1*, *TNFRSF19*, *CXCL6*, *LIFR*, *CXCL5*, *IL7*, *TSLP*, and *TNFSF15*, display poor survival rates respect to patients showing high levels of the aforementioned genes. This controversial scenario should be framed in the intricate multi-directional interplay among tumor cells and the diverse components of the TME. Moreover, studies focusing on the prognostic value of single genes in one cell type, rather than looking at the transcriptomic landscape of diverse cellular entities of a tumor mass, might not reflect the complexity existing within the TME.

## Conclusions

Overall, our findings indicate that inter-tumor heterogeneity in the gene expression profile of the breast and prostate microenvironment can be correlated with patient outcomes. The CAFs-related gene signatures here developed and validated might serve respectively as predictors of poor survival in breast and prostate tumor patients. Taken together, these data may potentially drive the management of the patients toward the establishment of tailored therapeutic strategies.

### Electronic supplementary material

Below is the link to the electronic supplementary material.

Supplementary Material 1

## Data Availability

Materials, additional data, and protocols described within the manuscript will be made available from the authors upon reasonable request. The raw data reported in this study are deposited in the GEO repository with accession number GSE269968.

## Abbreviations

AR: Androgen receptor
BCR: Biochemical recurrence
CAFs: Cancer-associated fibroblasts
DAPI: 4′,6-Diamidino-2-phenylindole dihydrochloride
DEGs: Differentially expressed genes
ECM: Extracellular matrix
EMP1: Epithelial membrane protein 1
EMP2: Epithelial membrane protein 2
EMPs: Epithelial membrane proteins
ER: Estrogen receptor
FAK: Focal adhesion kinase
FAP: Fibroblast activation protein α
FBS: Fetal bovine serum
FN1: Fibronectin 1
HER2: Human epidermal growth factor receptor
ITGA11: α11 Integrin
KEGG: The Kyoto Encyclopedia of Genes and Genomes
METABRIC: Molecular Taxonomy of Breast Cancer International Consortium
PR: Progesterone receptor
RNA-seq: RNA-sequencing
SPP1: Secreted phosphoprotein 1
SSE: Sum of square error
TCGA: The Cancer Genome Atlas
THBS1: Thrombospondin-1
TME: Tumor microenvironment
