# Supplementary material for: Cancer-associated fibroblasts (CAFs) gene signatures predict outcomes in breast and prostate tumor patients
Source: J Transl Med. 2024 Jun 27;22:597. doi: 10.1186/s12967-024-05413-2 (PMC11210052; doi:10.1186/s12967-024-05413-2)
Supplement: Supplementary file 1 — Supplementary Material 1 [file 12967_2024_5413_MOESM1_ESM.docx]

June 15, 2024

Dear Editor,

we are sending the manuscript entitled “Cancer-associated fibroblasts (CAFs) gene signatures predict

outcomes in breast and prostate tumor patients” by Talia et al., which has been revised in accordance with the suggestions made by the Reviewers.

We hope that our manuscript in its current form is now suitable for publication in Journal of Translational Medicine.

Thank you for your consideration. We look forward to hearing from you.

Sincerely,

Rosamaria Lappano
